# Prevalence and risk factors associated with multi-drug resistant organisms (MDRO) carriage among pediatric patients at the time of admission in a tertiary care hospital of a developing country. A cross-sectional study

**DOI:** 10.1186/s12879-021-06275-5

**Published:** 2021-06-09

**Authors:** Sonia Qureshi, Noshi Maria, Mohammad Zeeshan, Seema Irfan, Farah Naz Qamar

**Affiliations:** 1 Department of Pediatrics and Child Health, Aga Khan University Hospital, Karachi, Pakistan; 2 Department of Microbiology, Aga Khan University Hospital, Karachi, Pakistan

**Keywords:** Multidrug-resistant organisms, Carriage, Prevalence, Pediatric patients

## Abstract

**Background:**

The rise of Multidrug-resistant organisms (MDROs) poses a considerable burden on the healthcare systems, particularly in low-middle income countries like Pakistan. There is a scarcity of data on the carriage of MDRO particularly in the pediatrics population therefore, we aimed to determine MDRO carriage in pediatric patients at the time of admission to a tertiary care hospital in Karachi, Pakistan, and to identify the risk factors associated with it.

**Methods:**

A cross-sectional study conducted at the pediatric department of Aga Khan University Hospital (AKUH) from May to September 2019 on 347 children aged 1–18 years. For identification of MDRO (i.e., *Extended Spectrum Beta-Lactamase* (ESBL) producers, *Carbapenem Resistant Enterobacteriaceae* (CRE), *Vancomycin Resistant Enterococci* (VRE), M*ethicillin Resistant Staphylococcus aureus* (MRSA), Multidrug-resistant (MDR) *Acinetobacter species* and *MDR Pseudomonas aeruginosa*), nasal swabs and rectal swabs or stool samples were cultured on specific media within 72 h of hospitalization. Data was collected on a predesigned structured questionnaire on demographics, prior use of antibiotics for > 48 h in the last 6 months, history of vaccination in last 6 months, exposure to health care facility regardless of the time of exposure, ICU stay for > 72 h, and about the prior use of medical devices (urinary catheter, central venous lines etc.) in last 1 year. Statistical analysis was performed by Standard statistical software.

**Results:**

Out of 347 participants, 237 (68.3%) were found to be MDRO carriers. Forty nine nasal swabs from 346 children (14.2%) showed growth of MRSA. The majority of the stool/rectal swabs (*n* = 222 of 322; 69%) collected were positive for MDRO. The most isolated species were ESBL *Escherichia coli* 174/222 (78.3%) followed by ESBL *Enterobacter species* 37/222 (16.7%) and ESBL *Klebsiella pneumoniae* 35/222 (15.8%). On univariate analysis, none of the risk factors showed statistically significant association with MDRO carriage.

**Conclusion:**

Overall, a high prevalence of MDRO carriage was identified among admitted pediatric patients. Implementation of systematic screening may help to identify true burden of MDROs carriage in the health care settings.

## Background

The rapidly emerging multidrug-resistant organisms (MDROs) associated infections with meager and scarce availability of better therapeutic options are among the prioritized global health concerns [1]. Based on drug resistance patterns, MDROs are categorized into multidrug-resistant (MDR), extensively drug-resistant (XDR) and pan drug-resistant (PDR) pathogenic bacteria.” MDROs are non-susceptible to at least one agent in three or more antimicrobial categories, XDR bacteria are non-susceptible to at least one agent in all but two or fewer antimicrobial categories, and PDR bacteria are non-susceptible to all agents in all antimicrobial categories [2].

The spectrum of hospital acquired multidrug bacteria include *Staphylococcus aureus*, *Enterococci species*, members of family *Enterobacteriaceae, Pseudomonas aeruginosa,* and A*cinetobacter species* [3]. Although the prevalence of these pathogens varies geographically, temporally and by healthcare settings, no institution is without the menace from these organisms rendering the standard of public health in many regions of the world equivalent to the pre-antibiotic era [4]. Besides heightening the risk of morbidity and mortality in vulnerable individuals, other negative repercussions of MDROs include longer hospitalization and higher medical costs [5, 6].

Over the past two decades, the strains of MDROs have quadrupled particularly among children and cause serious bacterial infections (SBIs) including bacteremia, meningitis, bone and joint infections, urinary tract infections (UTI), gastroenteritis, and skin or soft tissue infections [7, 8]. National Healthcare Safety Network (NHSN) reported that MDROs cause infections in ~ 5–10% hospitalized children [9]. Studies reported a 4% annual increase in multidrug resistant strains of *P. aeruginosa* [10] and a significant rise in carbapenem resistance in *Enterobacteriaceae strains* [11, 12]. The cases of *vancomycin resistant enterococci* infections in hospitalized children also increased from 53 per million in 1997 to 120 per million in 2012.

Acquisition of these drug resistant bacteria in infants is either through the mother or from the community [13]. A Report from a tertiary care hospital of Tunisia showed 8.9% of their pediatric patients’ colonization with at least one drug resistant bacteria before and during hospital admission [14]. In the USA approximately 4% of children admitted to a Pediatric Intensive Care Unit (PICU) were found to be carriers of MDR *Enterobacteriaceae* [15]. Colonization of MDR *Streptococcus pneumoniae* was reported 5% among healthy Thai children [16].

The colonization locations include body sites normally inhabited by indigenous human microbiota or in chronic wounds, renal calculi, devitalized bones, and indwelling foreign bodies [3]. Left unchecked, these carriers serve as reservoirs and vehicles in the transmission of MDROs; raise the burden of MDRO infections in health care particularly in intensive and acute care settings [17]. There are multiple ways to detect the MDRO carriers like from rectal and nasal swabs. It has been shown that the sensitivity of detecting microorganisms like MRSA can be increased by taking cultures from multiple sites like the nose, pharynx, and skin instead of just one site. Moreover, using skin sponges instead of skin swabs can also increase the detection sensitivity by covering a larger skin area [18].

There are studies regarding MDROs associated infections in Pakistani adults [19–22], but relatively scarce data is available of the pediatric population. Moreover, the focus of previous studies was on the acquisition of MDROs during hospital stay rather than identification of MDRO carriage at the time of admission [23]. We hypothesized that there is an association between MDRO carriage and risk factors such as prior use of antibiotic, prior hospitalization, or exposure to health care facility in children. Therefore, the objective of our study is to determine the prevalence of carriage of specific MDROs in pediatric patients admitted to a tertiary care center in Karachi and the risk factors associated with the carriage. This data will help to propose and develop guidelines for appropriate selection and usage of antibiotics and will impact infection control practices and antimicrobial stewardship efforts.

## Methods

### Patients and Study setting

We conducted a cross-sectional study including all pediatric patients aged 1–18 years admitted to the pediatric ward at the Aga Khan University Hospital (AKUH), Pakistan, from May to September 2019. Participants were screened for identification of MDRO carriers including MRSA, VRE, CRE and ESBL-producing gram negative bacteria within 72 h of admittance. Patients with a documented history of culture proven MDRO infection or carriage during the previous 12 months of admission, diagnosed cases of primary or secondary immunodeficiency, and those who refused to participate in the study were excluded.

This study was approved by the Ethical Review Committee (ERC) of AKUH (2019–0653-2209). All methods including enrolment of patients, data collection, microbiological and statistical analysis were in accordance with the guidelines and regulations of institutional ERC. The sample size was calculated by OpenEpi. Assuming a rate of MDRO carriers in the general population as 30%, 5% bound on the error, 5% significance level, the maximum sample size was 323. Sample size was also calculated for associated risk factors such as prior use of antibiotics, prior hospitalization, ICU stay etc. by taking frequency of these factors among non-MDRO carriers between 30% and 50%, 80% power, 5% significance level, and ratio of-MDRO carriers to non-MDRO carriers as 1:2, with a prevalence ratio (PR) of 2.00 and 5% attrition, the sample size estimated was at least 323.

### Data collection method

Data was collected aftertaking consent from parents or guardians of patients, on demographics, prior use of antibiotics for > 48 h in the last 6 months, history of vaccination in last 6 months, exposure to health care facility regardless of the time of exposure, ICU stay for more than 72 h, and about the prior use of medical devices (urinary catheter, central venous lines etc.) in last 1 year. We cultured the nasal swab for MRSA, and either rectal swab or stool specimen for ESBL *Enterobacteriaceae*, carbapenem resistant *Enterobacteriaceae*, vancomycin resistant *enterococci, MDR* Acinetobacter species, and *MDR P. aeruginosa* within 72 h of hospitalization for the detection of these pathogens. Patient was identified as MDRO carrier if a (nasal, rectal or stool) was positive, and the patient was without systemic infection caused by these organisms.

### Microbiological analysis

For the detection of VRE, rectal swabs/stool specimens were streaked directly onto selective agar plates and incubated aerobically for 24 h. Isolated colonies of *enterococci* were confirmed as *E. faecalis* or *E. faecium* by biochemical test and API strep kit test. These isolates were confirmed as vancomycin resistant by vancomycin disc on SB-Mueller-Hinton agar (SBMHA) plate and isolated on 6mcg/ml vancomycin plate with negative and positive control.

Swabs were also inoculated into a broth medium containing 6 mcg/ml vancomycin (Enterococcosel). Broth with color change from amber to black after 24–48 h incubation at 37 °C was then sub-cultured on blood agar and selective VRE media and incubated overnight at 37 °C. For further confirmation of isolated colonies API strep kit test and vancomycin disc on Mueller Hinton Agar (MHA) media was performed.

For identification of MRSA, nasal swabs plated directly onto sheep blood agar (SBA) and mannitol salt agar (MSA) and incubated aerobically for 24 h in a CO_2_ incubator. A Swab was also inoculated into 2.5% NaCl BHI (Brain Heart Infusion) broth and after incubation at 37 °C for 24 h; it is inoculated onto colistin nalidixic acid agar (CNA), MSA, and SBA plates. The plates were incubated at 37 °C for 24 h. Morphologically resembling *S. aureus* was confirmed by tube coagulase (for secreted extracellular coagulase), DNase agar, and phenolphthalein phosphate agar. Broth or saline suspension of colonies confirmed as *S. aureus* was prepared and adjusted to achieve turbidity of 0.5 McFarland standards. Sensitivity testing carried out by disc diffusion method against cefoxitin 30 mcg disc susceptibility breakpoints for cefoxitin against *S. aureus* and was interpreted using Clinical and Laboratory Standards Institute (CLSI) guidelines.

Rectal swabs and stool specimens were inoculated into BHI broth and after overnight incubation at 35 ± 2 °C with ambient air, vortexed, and subcultured 100 μl onto MacConkey and CNA agar plate with vancomycin disc placed on 1st streak of CNA. Followed by overnight incubation, all gram negative bacilli were identified by conventional biochemical identification method that includes oxidase test, triple sugar iron (TSI) agar, Sulphide indole motility (SIM) agar, citrate agar, urea agar, Pseudomonas agar P, and Pseudomonas agar F. Identification of ESBL producing *Enterobacteriaceae* was performed by disc diffusion method by using combination of clavulanic acid (10 μg) and ceftazidime (30 μg). Both discs were placed on Muller Hinton agar plates which were earlier swabbed by respective culture and incubated for 24 h at 37 °C. More than 5 mm increase in the zone diameter for ceftazidime-clavulanic acid was considered positive ESBL production. Phenotypic disc diffusion method with Imipenem disc (10 μg) on Muller Hinton agar plates and incubated for 24 h at 37 °C were used for detection of carbapenem resistance in *Enterobacteriaceae* (zone size ≤19 mm), *Acinetobacter species* (zone size ≤18 mm), and *P. aeruginosa* (Zone size ≤15 mm). Identification of XDR *S.* Typhi was done using conventional biochemical methods i.e., TSI, SIM agar, and confirmed with the help of serotyping (BD Difco Salmonella O Antiserum Factor 9) and API 20E (Biomerieux).

### Statistical analysis

Statistical analysis was performed on Stata version 12.0. Descriptive statistics were reported as frequencies with percentages for categorical variables like gender, prior use of antibiotics, prior hospitalization, etc., and mean ± SD was reported for age (years). Univariate analysis was performed by Cox proportional hazards algorithm, *p*-value ≤0.25 was considered significant. The results are reported as crude PR with 95% confidence intervals.

## Results

**Fig. 1:**
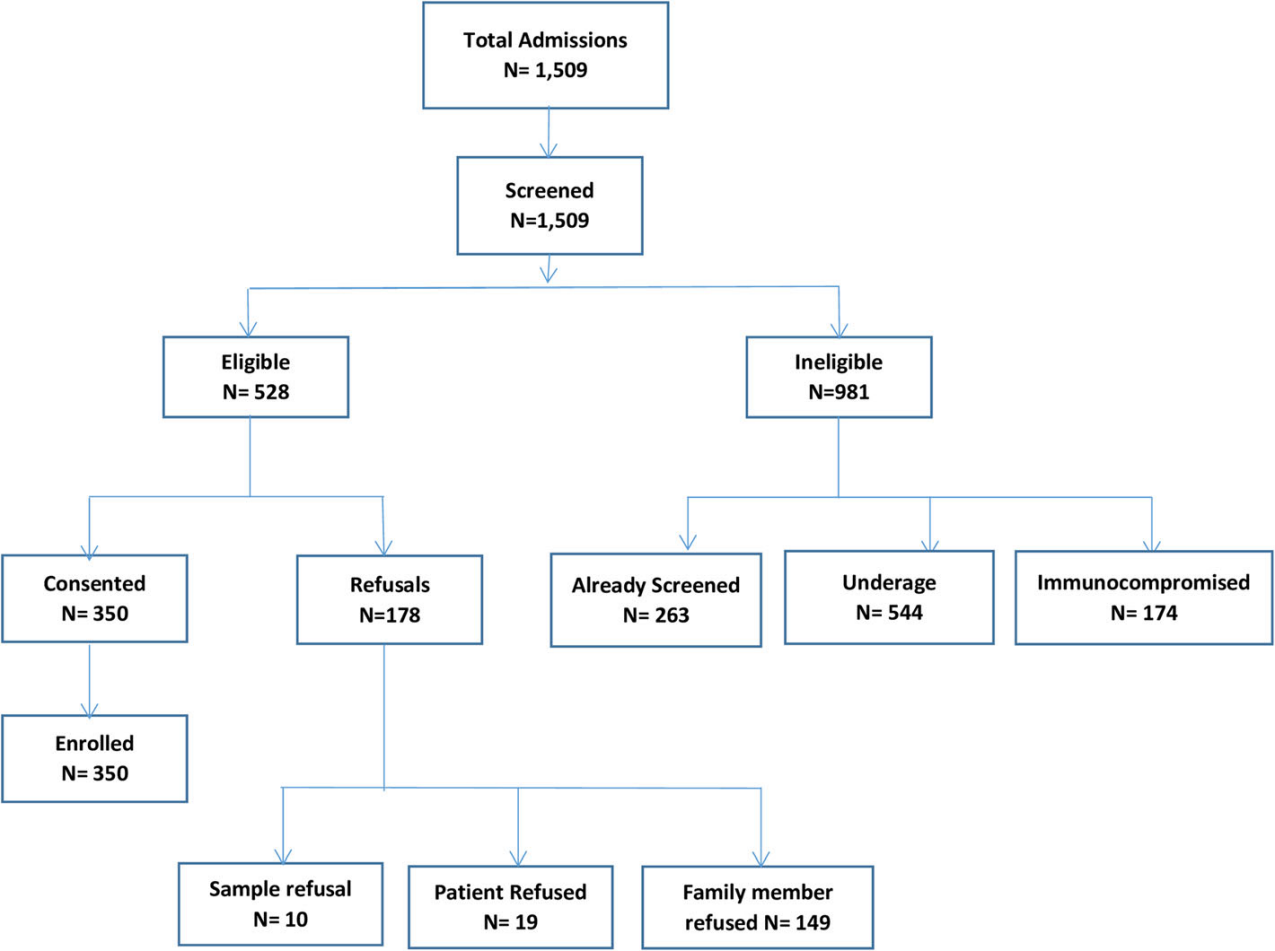
Study flow (Uploaded as a separate PDF file)

**Fig. 2:**
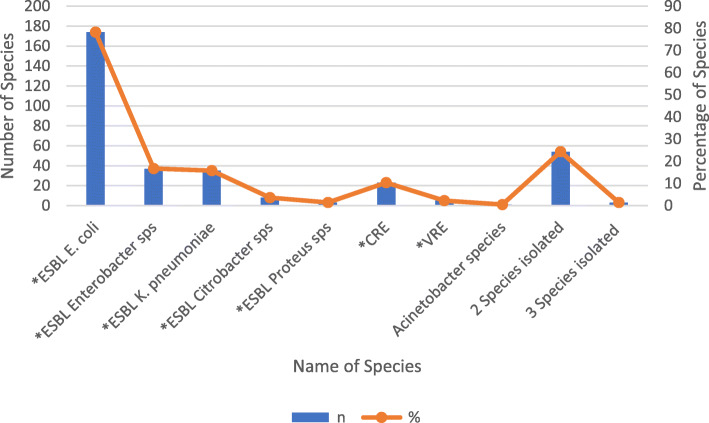
Proportion of various microorganisms identified from positive stool/rectal swab culture (N = 222) (Uploaded as a separate PDF file)

A total of 1509 patients were admitted during the study period and screened for participation, of which 528 (35%) fulfilled the eligibility criteria. 350 (66.3%) consented to participate and were enrolled (Fig. 1). Three patients were excluded from the analysis as they refused to provide samples. The details of demographic and clinical characteristics are provided in Table 1**.** The common underlying diagnosis of the participants were 48.7% infections followed by 17.3% gastrointestinal and 12.7% respiratory illnesses (Table 2). From 322 stool/rectal swab specimens collected, 222 (68.9%) were positive for MDRO. The most isolated species were ESBL *E. coli* 174/222 (78.3%) followed by *ESBL Enterobacter species* 37/222 (16.7%) and ESBL *K. pneumoniae* 35/222 (15.8%) (Fig. 2). None of the risk factors was statistically significant on univariate analysis (Table 3).

**Table 1:** Descriptive statistics of study participants admitted in a tertiary care hospital (N = 347)

Variables			n (%)
Age (years), Mean ± SD			6.7 ± 4.9
Gender	Male		194 (55.9)
Unit of care	Ward	^a^SCU	3 (0.9)
		General	251 (72.3)
		Semiprivate	27 (7.8)
		Private Wing	23 (6.6)
	^a^PICU		43 (12.4)
Prior antibiotic use for > 48 h in the last 6 months			70 (20.2)
History of vaccination in the last 6 months			45 (13.0)
Prior health care facility exposure in the last 1 year			320 (92.2)
Past ICU stay > 72 h in the last 1 year			8 (2.3)
Prior use of medical devices at any time in the last 1 year			1 (0.3)
Stool culture positive for ^a^MDRO			41/65 (63.1)
Rectal swab culture positive for ^a^MDRO			181/257 (70.4)
Nasal swab culture positive for ^a^MRSA			49/346 (14.2)
^a^MDRO carriers			237 (68.3)

^a^*SCU* Special Care Unit, *PICU* Pediatric Intensive Care Unit, *MDRO* Multi Drug Resistant Organism, *MRSA* Methicillin-Resistant Staphylococcus aureus

**Table 2:** Descriptive characteristics of underlying diagnosis in children with MDRO colonization at the time of admission in a tertiary care hospital (N = 347)

Diagnosis		***n*** (%)
**Gastrointestinal Illnesses (*n*** **= 60)**		
Acute Gastritis and peptic ulcer		14 (23.3)
Gastroenteritis		39 (68.3)
Constipation		3 (5.0)
Dysphagia		1 (1.7)
Lower GI bleed		1 (1.7)
**Respiratory illness and Allergies (*n*** **= 44)**		
Upper Respiratory Tract Infection		7 (15.9)
Bronchiolitis		3 (6.8)
Bronchopneumonia		14 (31.8)
Aspiration pneumonia		1 (2.3)
Acute Exacerbation of Asthma		14 (31.8)
Urticarial rash		5 (11.4)
**Infectious Diseases (*n*** **= 169)**		
Cholera		1 (0.6)
Dysentery		6 (3.5)
Urinary Tract Infection		5 (3.0)
Viral Fever		3 (1.8)
Group C streptococcal sepsis		1 (0.6)
Herpetic gingivitis		1 (0.6)
Dengue Fever		8 (4.7)
Malaria		2 (1.2)
Typhoid Fever (N = 107)	Presumed Typhoid Fever	54 (50.5)
	XDR Typhoid Fever	38 (35.5)
	Typhoid fever	15 (14.0)
Acute Viral hepatitis		11 (6.5)
Invasive fungal sinusitis		1 (0.6)
Skin, soft tissue and bone infections (N = 12)	^a^Abscess	6 (50.0)
	Cellulitis	5 (41.7)
	Right hip osteomyelitis	1 (8.3)
Meningitis (N = 7)	Viral	3 (42.8)
	Bacterial	1 (14.3)
	Tuberculosis	1 (14.3)
	Suspected	2 (28.6)
^b^Others		4 (2.4)
^**c**^**Cardiovascular diseases (*n*** **= 6)**		
**Neuropsychiatric diseases (*n*** **= 19)**		
Seizure disorder		2 (10.5)
Febrile fits		9 (47.3)
Syncope		2 (10.5)
Suspected Psychiatric illness		2 (10.5)
^d^Others		4 (21.0)
**Endocrine and Metabolic Diseases (*n*** **= 12)**		
Osteogenesis Imperfecta for zolendronate infusion		3 (25.0)
HMDPC Syndrome for EDTA chelation		3 (25.0)
^e^Others		6 (50.0)
**Autoimmune Disease (*n*** **= 7)**		
Celiac Disease		2 (28.5)
Suspected Inflammatory Bowel Disease		1 (14.3)
Autoimmune Vasculitis		1 (14.3)
Systemic lupus erythematosus (SLE)		2 (28.6)
Polyarthritis for workup		1 (14.3)
**Trauma and Accidents (*n*** **= 4)**		
Concussion injury		1 (25.0)
History of Fall for observation		2 (50.0)
Drowning		1 (25.0)
**Miscellaneous (*n*** **= 26)**		
Pancreatitis		3 (11.5)
Iron deficiency		2 (7.7)
Nephrotic syndrome		5 (19.2)
Nonspecific abdominal pain		2 (7.7)
^f^Others		14 (53.8)

*GI* Gastrointestinal, *XDR* Extensively drug resistant, *ASD* Atrial Septal Defect, *PDA* Patent Ductus Arteriosus, *HMDPC* Hypermanganesemia with Dystonia, Polycythemia and Cirrhosis, *EDTA* Ethylenediaminetetraacetic acid

^*a*^*Liver abscess (MRSA: Methicillin-Resistant Staphylococcus aureus* (1)*, amebic liver abscess* (1)*), Chin abscess (MRSA: Methicillin-Resistant Staphylococcus aureus), Chest wall abscess (MRSA: Methicillin-Resistant Staphylococcus aureus)*

^*b*^*Abdominal Tuberculosis* (1)*, Disseminated Tuberculosis* (1)*, Left pyelitis* (1)*, Post viral myalgia* (1)

^*c*^*ASD repair* (1)*, PDA device closure* (2)*, Complex Congenital Heart Disease for Right heart catheterization* (2)*, Postural hypotension* (1)

^*d*^*Acute Disseminated Encephalomyelitis* (1)*, Migraine* (1)*, Intraventricular Hemorrhage* (1)*, Developmental delay for workup* (1)

^*e*^*Insulin Dependent Diabetes Mellitus* (1)*, Hypoglycemia* (1)*, hypocalcemic fits* (1)*, Fructose 1,6 bisphosphatase Deficiency* (1)*, Ketotic hypoglycemia* (1)*, Maple Syrup Disease with Leucine Toxicity* (1)

^*f*^*Excessive crying for observation* (1)*, Scorpion bite* (1)*, Spondylodiscitis (L4–5)* (1)*, Muscular spasm* (1)*, Inderal Overdose* (1)*, Ultrasound guided liver biopsy* (1)*, Impacted ear wax* (1)*, Obstructive jaundice* (1)*, Chronic Liver Disease for band ligation* (1)*, Posterior fossa mass* (1)*, Suspected Zinc deficiency* (1)*, Temporomandibular Joint Disorder* (1)*, Bleeding Disorder* (1)*, Breath holding spells* (1)

**Table 3:** Unadjusted analysis for clinical features associated with MDRO carriage in children at the time of admission in a tertiary care hospital, Karachi

Variables		Crude PR	95%CI	***p-***value
Age (years)		0.99	0.97, 1.02	0.91
Gender	Male	0.96	0.74, 1.23	0.74
	^a^Female	1.00		
Unit of care	PICU	0.87	0.58, 1.31	0.51
	^a^Ward	1.00		
Prior antibiotic use for > 48 h in the last 6 months	Yes	1.03	0.75, 1.41	0.85
	^a^No	1.00		
History of vaccination in the last 6 months	No	0.95	0.66, 1.39	0.81
	^a^Yes	1.00		
Prior health care facility exposure in the last 1 year	Yes	1.09	0.67, 1.79	0.72
	^a^No	1.00		
Past ICU stay > 72 h in the last 1 year	Yes	0.54	0.17, 1.70	0.29
	^a^No	1.00		
Number of health care facility exposure	Multiple	1.01	0.72, 1.41	0.97
	^a^Single	1.00		

^a^Reference Category

## Discussion

In this study, we did a systematic screening of Pakistani children to assess MDRO carriage amongst them. The overall MDRO carriage in our study was found to be very high i.e. 68.3% as compared to China where overall MDRO prevalence is 36% [24]. The proportion of MRSA carriage was also elevated (14.2%) in contrast to China where MRSA carriage is 6.4% (28). Out of 322 stool/rectal swab specimens, 68.9% were positive for MDRO. The most isolated species were ESBL *E. coli* (78.3%), ESBL *enterobacter species* (16.7%), and ESBL *K. pneumoniae* (15.8%). These observed percentages were also higher than those previously reported from other countries such as Guinea-Bissau (ESBL *E. coli* 47.7% and *K. pneumoniae* 15.8%), Madagascar (ESBL *E. coli* and *K. pneumoniae* 36.9%), Lebanon (ESBL *Enterobacteriaceae* 24.9%), and Thailand (ESBL *E. coli* 67.5% and *K. pneumoniae* 19.4%) [25–28].

The risk factors reportedly associated with MDRO carriage include age, increased usage of antibiotics [29], prior exposure to hospital settings; especially to the ICU [14] and the total length of stay in hospital settings [30]. In our study, none of these risk factors was found to be associated with MDRO carrier. One study reported that the use of cephalosporin within the preceding 4 months, age less than 2 years, and having a sibling colonized with resistant *Streptococcus pneumoniae* (*S. pneumoniae*) were significantly associated with carriage of resistant *S. pneumoniae* [31]. Another study showed no association of MDRO carriage with age and gender but a significant association of MDRO carriage with use of antibiotics in the last 30 days, hospitalization in the past 2 years, and intrahospital transfer [32]. On the contrary, findings reported from Thailand were less conclusive and did not find any significant association of MDRO carriage with antibiotic use in the past 30 days [16]. Similarly, there are further studies that were inconclusive for any association of MDRO carriage with age, gender, previous antibiotic use, or hospitalization [14, 32].

Poor hygiene and sanitary conditions likely contribute as a factor for the high percentage of MDRO carrier in our region. Our findings also raise the concern of asymptomatic carriage of MDRO in the community. This increases the risk of dissemination of resistance genes by human-to-human transmission or by contamination of the environment [33]. Although, antibiotic history was not found to be significantly associated with MDRO carriage in our study, however, literature mentions it as a factor significantly associated with increased rates of MDROs in the community, including young infants [34]. Other risk factors contributing to the high percentage of MDRO carriage include self-prescription and inappropriate usage of antibiotics [35, 36], nescience and lack of effective antimicrobial stewardship programs in hospitals [37] and scarcity of physicians specialized in pediatric or adult infectious diseases [38]. Our results might increase the awareness among physicians about the high prevalence of MDROs in the pediatric age group and young infants. It might also encourage all healthcare providers to contribute stopping the spread of MDRO by using the appropriate antibiotics in the inpatient and outpatient settings.

Our study is not free of limitations. The current data from a single-center study may not reflect the prevalence of MDRO in Pakistan as a whole. Therefore, a multicenter study is needed to compare different healthcare facilities across urban and rural settings of our region to get a better understanding of the situation. As this study was observational, there might be a recall bias which may explain the lack of a significant association between prior antibiotic use and MDRO carriage. Furthermore, the narrow time frame of this study restricted how much the effect of climatic conditions on MDRO carriage could be researched.

Regardless of all these limitations, the current study is of great importance as to the best of our knowledge it is the first study of our region that presents data on MDRO carriage in pediatric patients.

## Conclusion

High prevalence of MDRO carriers in children poses a great public health concern, especially in developing countries like Pakistan where the health care system is already over-burdened. Our findings call for the systematic screening of patients at the time of admission as well as in communities to identify asymptomatic carriers who act as disseminators, in order to find out true burden of MDROs. There should be more targeted epidemiologic and genotypic studies to provide insights on MDRO acquisition and carriage in children.

## Data Availability

The data supporting the results of the current study are contained within the manuscript.

## Abbreviations

AKUH: Aga Khan University Hospital
ASD: Atrial Septal Defect
BHI: Brain Heart Infusion
CLSI: Clinical and Laboratory Standards Institute
CNA: Colistin nalidixic acid agar
CRE: *Carbapenem Resistant Enterobacteriaceae*
DIPHE: Department of infection prevention and health epidemiology
EDTA: Ethylenediaminetetraacetic acid
ERC: Ethical review committee
ESBL: *Extended Spectrum Beta-Lactamase*
GI: Gastrointestinal
GNR: Gram negative rods
HMDPC: Hypermanganesemia with Dystonia, Polycythemia, and Cirrhosis
ICU: Intensive care unit
IP: Infection practitioner
MDR: Multidrug-resistant
MDRO: Multidrug-resistant organisms
MHA: Mueller Hinton Agar
MRSA: M*ethicillin Resistant Staphylococcus aureus*
MSA: Mannitol salt agar
NHSN: National Healthcare Safety Network
PDA: Patent Ductus Arteriosus
PDR: Pan drug-resistant
PICU: Pediatric Intensive Care Unit
PR: Prevalence ratios
SBA: Sheep blood agar
SBIs: Serious bacterial infections
SBMHA: SB-Mueller-Hinton agar
SCU: Special Care Unit
SD: Standard Deviation
SIM: Sulphide indole motility agar
SLE: Systemic lupus erythematosus
TSI: Triple sugar iron agar
UTI: Urinary tract infections
VRE: *Vancomycin Resistant Enterococci*
XDR: Extensively drug-resistant

## References

[1] Laxminarayan R, Van Boeckel T, Frost I, Kariuki S, Khan EA, Limmathurotsakul D (2020). The lancet infectious diseases commission on antimicrobial resistance: 6 years later. Lancet Infect Dis.

[2] Magiorakos AP, Srinivasan A, Carey RB, Carmeli Y, Falagas ME, Giske CG (2012). Multidrug-resistant, extensively drug-resistant and pandrug-resistant bacteria: an international expert proposal for interim standard definitions for acquired resistance. Clin Microbiol Infect.

[3] Tosh PK, McDonald LC (2012). Infection control in the multidrug-resistant era: tending the human microbiome. Clin Infect Dis.

[4] Arias CA, Murray BE (2009). Antibiotic-resistant bugs in the 21st century--a clinical super-challenge. N Engl J Med.

[5] Rosenberger LH, Hranjec T, Politano AD, Swenson BR, Metzger R, Bonatti H (2011). Effective cohorting and "superisolation" in a single intensive care unit in response to an outbreak of diverse multi-drug-resistant organisms. Surg Infect.

[6] Morales E, Cots F, Sala M, Comas M, Belvis F, Riu M (2012). Hospital costs of nosocomial multi-drug resistant Pseudomonas aeruginosa acquisition. BMC Health Serv Res.

[7] Yusef D, Jahmani T, Kailani S, Al-Rawi R, Khasawneh W, Almomani M (2019). Community-acquired serious bacterial infections in the first 90 days of life: a revisit in the era of multi-drug-resistant organisms. World J Pediatr.

[8] Folgori L, Bernaschi P, Piga S, Carletti M, Cunha FP, Lara PH (2016). Healthcare-associated infections in pediatric and neonatal intensive care units: impact of underlying risk factors and antimicrobial resistance on 30-day case-fatality in Italy and Brazil. Infect Control Hosp Epidemiol.

[9] Hidron AI, Edwards JR, Patel J, Horan TC, Sievert DM, Pollock DA (2008). NHSN annual update: antimicrobial-resistant pathogens associated with healthcare-associated infections: annual summary of data reported to the National Healthcare Safety Network at the Centers for Disease Control and Prevention, 2006-2007. Infect Control Hosp Epidemiol.

[10] Logan LK, Gandra S, Mandal S, Klein EY, Levinson J, Weinstein RA (2017). Multidrug- and Carbapenem-resistant Pseudomonas aeruginosa in children, United States, 1999-2012. J Pediatric Infect Dis Soc.

[11] Logan LK, Renschler JP, Gandra S, Weinstein RA, Laxminarayan R, Centers for disease C (2015). Carbapenem-resistant Enterobacteriaceae in children, United States, 1999-2012. Emerg Infect Dis.

[12] Meropol SB, Haupt AA, Debanne SM (2018). Incidence and outcomes of infections caused by multidrug-resistant Enterobacteriaceae in children, 2007-2015. J Pediatric Infect Dis Soc.

[13] DePorre AG, Aronson PL, McCulloh RJ (2017). Facing the ongoing challenge of the febrile young infant. Crit Care.

[14] Tfifha M, Ferjani A, Mallouli M, Mlika N, Abroug S, Boukadida J (2018). Carriage of multidrug-resistant bacteria among pediatric patients before and during their hospitalization in a tertiary pediatric unit in Tunisia. Libyan J Med.

[15] Suwantarat N, Logan LK, Carroll KC, Bonomo RA, Simner PJ, Rudin SD (2016). The prevalence and molecular epidemiology of multidrug-resistant Enterobacteriaceae colonization in a pediatric intensive care unit. Infect Control Hosp Epidemiol.

[16] Thummeepak R, Leerach N, Kunthalert D, Tangchaisuriya U, Thanwisai A, Sitthisak S (2015). High prevalence of multi-drug resistant Streptococcus pneumoniae among healthy children in Thailand. J Infect Public Health.

[17] Saiman L, Cronquist A, Wu F, Zhou J, Rubenstein D, Eisner W (2003). An outbreak of methicillin-resistant Staphylococcus aureus in a neonatal intensive care unit. Infect Control Hosp Epidemiol.

[18] Lee CS, Montalmont B, O'Hara JA, Syed A, Chaussard C, McGaha TL (2015). Screening for methicillin-resistant Staphylococcus aureus colonization using sponges. Infect Control Hosp Epidemiol.

[19] Das JK, Hasan R, Zafar A, Ahmed I, Ikram A, Nizamuddin S (2018). Trends, associations, and antimicrobial resistance of salmonella Typhi and Paratyphi in Pakistan. Am J Trop Med Hyg.

[20] Jabeen K, Bhawan Mal P, Khan E, Chandio S, Jacobsson S, Unemo M (2016). Antimicrobial resistance and Neisseria gonorrhoeae multiantigen sequence typing (NG-MAST) genotypes in N. gonorrhoeae during 2012-2014 in Karachi, Pakistan. BMC Infect Dis.

[21] Khan MS, Durrance-Bagale A, Legido-Quigley H, Mateus A, Hasan R, Spencer J (2019). 'LMICs as reservoirs of AMR': a comparative analysis of policy discourse on antimicrobial resistance with reference to Pakistan. Health Policy Plan.

[22] Nahid F, Khan AA, Rehman S, Zahra R (2013). Prevalence of metallo-beta-lactamase NDM-1-producing multi-drug resistant bacteria at two Pakistani hospitals and implications for public health. J Infect Public Health.

[23] Begum S, Hasan F, Hussain S, Ali SA (2013). Prevalence of multi drug resistant Acinetobacter baumannii in the clinical samples from tertiary Care Hospital in Islamabad. Pakistan Pak J Med Sci.

[24] Ma X, Wu Y, Li L, Xu Q, Hu B, Ni Y (2015). First multicenter study on multidrug resistant bacteria carriage in Chinese ICUs. BMC Infect Dis.

[25] Isendahl J, Turlej-Rogacka A, Manjuba C, Rodrigues A, Giske CG, Naucler P (2012). Fecal carriage of ESBL-producing *E. coli* and *K. pneumoniae* in children in Guinea-Bissau: a hospital-based cross-sectional study. PLoS One.

[26] Andriatahina T, Randrianirina F, Hariniana ER, Talarmin A, Raobijaona H, Buisson Y (2010). High prevalence of fecal carriage of extended-spectrum beta-lactamase-producing Escherichia coli and Klebsiella pneumoniae in a pediatric unit in Madagascar. BMC Infect Dis.

[27] Hijazi SM, Fawzi MA, Ali FM, Abd El Galil KH (2016). Multidrug-resistant ESBL-producing Enterobacteriaceae and associated risk factors in community infants in Lebanon. J Infect Dev Ctries.

[28] Kiddee A, Assawatheptawee K, Na-Udom A, Boonsawang P, Treebupachatsakul P, Walsh TR (2019). Risk factors for extended-Spectrum beta-lactamase-producing Enterobacteriaceae carriage in patients admitted to intensive care unit in a tertiary Care Hospital in Thailand. Microb Drug Resist.

[29] Murray MT, Beauchemin MP, Neu N, Larson EL (2019). Prior antibiotic use and acquisition of multidrug-resistant organisms in hospitalized children: a systematic review. Infect Control Hosp Epidemiol.

[30] Buke C, Armand-Lefevre L, Lolom I, Guerinot W, Deblangy C, Ruimy R (2007). Epidemiology of multidrug-resistant bacteria in patients with long hospital stays. Infect Control Hosp Epidemiol.

[31] Samore MH, Magill MK, Alder SC, Severina E, Morrison-De Boer L, Lyon JL (2001). High rates of multiple antibiotic resistance in Streptococcus pneumoniae from healthy children living in isolated rural communities: association with cephalosporin use and intrafamilial transmission. Pediatrics..

[32] Tarchouna M, Ferjani A, Tilouche S, Marzouk M, Bouguila J, Boughamoura L (2014). Screening at admission for carrier prevalence of multidrug-resistant organisms in a pediatric unit. Am J Infect Control.

[33] Bebell LM, Muiru AN (2014). Antibiotic use and emerging resistance: how can resource-limited countries turn the tide?. Glob Heart.

[34] Baig MT, Sial AA, Huma A, Ahmed M, Shahid U, Syed N (2017). Irrational antibiotic prescribing practice among children in critical care of tertiary hospitals. Pak J Pharm Sci.

[35] Ali M, Abbasi BH, Ahmad N, Fazal H, Khan J, Ali SS (2020). Over-the-counter medicines in Pakistan: misuse and overuse. Lancet..

[36] Saleem Z, Saeed H, Hassali MA, Godman B, Asif U, Yousaf M (2019). Pattern of inappropriate antibiotic use among hospitalized patients in Pakistan: a longitudinal surveillance and implications. Antimicrob Resist Infect Control.

[37] Hayat K, Rosenthal M, Gillani AH, Zhai P, Aziz MM, Ji W, et al. Perspective of Pakistani physicians towards hospital antimicrobial stewardship programs: a multisite exploratory qualitative study. Int J Environ Res Public Health. 2019;16(9):1565. 10.3390/ijerph16091565 PMC6539566 31060262

[38] WHO Global Code of Practice on International Recruitment of Health Personnel. 2011.

